# Plant-Rich Field Margins Influence Natural Predators of Aphids More Than Intercropping in Common Bean

**DOI:** 10.3390/insects13070569

**Published:** 2022-06-23

**Authors:** Baltazar J. Ndakidemi, Ernest R. Mbega, Patrick A. Ndakidemi, Steven R. Belmain, Sarah E. J. Arnold, Victoria C. Woolley, Philip C. Stevenson

**Affiliations:** 1 Department of Sustainable Agriculture, School of Life Sciences and Bioengineering, The Nelson Mandela African Institution of Science and Technology, P.O. Box 447, Arusha 23218, Tanzania; ernest.mbega@nm-aist.ac.tz (E.R.M.); patrick.ndakidemi@nm-aist.ac.tz (P.A.N.); s.e.j.arnold@greenwich.ac.uk (S.E.J.A.); 2 Natural Resources Institute, University of Greenwich, Chatham Maritime, Kent ME4 4TB, UK; s.r.belmain@greenwich.ac.uk (S.R.B.); v.woolley291@gmail.com (V.C.W.); p.c.stevenson@greenwich.ac.uk (P.C.S.); 3 Royal Botanic Gardens, Kew, Kew Green, Richmond, Surrey TW9 3AE, UK

**Keywords:** natural enemies, predators, parasitoids, conservation biological control, field margin, *Phaseolus vulgaris*

## Abstract

**Simple Summary:**

Field margin plants are important in providing resources for natural enemies (NEs) and improving biological control of crop pests. However, the use of field margin plants for biological control particularly of important common bean pests is understudied in smallholder farming systems of sub-Saharan Africa (SSA). We evaluated the potential of field margin plants with respect to intercropping systems in common bean fields to enhance the population of NEs of common bean pests. We observed a high assemblage of important NEs of common bean pests for some insect taxa with minimal impact of intercropping on NEs. Field margin plants could be managed to provide a wide range of resources to NEs and therefore biological control of common bean pests.

**Abstract:**

Field margins support important ecosystem services including natural pest regulation. We investigated the influence of field margins on the spatial and temporal distribution of natural enemies (NEs) of bean pests in smallholder farming systems. We sampled NEs from high and low plant diversity bean fields using sweep netting and coloured sticky traps, comparing monocropped and intercropped farms. NEs collected from within crops included predatory bugs, lacewings, predatory flies, parasitic flies, parasitic wasps, lady beetles, and a range of other predatory beetles; with the most dominant group being parasitic wasps. Overall, high plant diversity fields had a higher number of NEs than low-diversity fields, regardless of sampling methods. The field margin had a significantly higher number of lacewings, parasitic wasps, predatory bugs, syrphid flies, and other predatory beetles relative to the crop, but beneficial insects were collected throughout the fields. However, we observed marginally higher populations of NEs in intercropping than in monocropping although the effect was not significant in both low and high plant diversity fields. We recommend smallholder farmers protect the field margins for the added benefit of natural pest regulation in their fields.

## 1. Introduction

Common bean (*Phaseolus vulgaris* L.) is one of the most important legume crops in sub-Saharan Africa (SSA) for the provision of proteins, vitamins, energy, and macronutrients [1,2] and due to its ability to fix nitrogen which contributes to soil fertility [2]. However, common bean production is constrained by insect pests [3]. One of the most serious is the black bean aphid *Aphis fabae* Scopoli which causes yield losses in the common bean of up to 90% in East Africa [4,5,6,7]. *A. fabae* also transmit plant diseases including bean common mosaic necrosis virus (BCMNV), bean common mosaic virus (BCMV) and cucumber mosaic virus (CMV) [8]. While many smallholder farmers do not apply any control measures because of the high cost and lack of knowledge [9,10], others apply synthetic pesticides indiscriminately [11]. This approach is not sustainable and there are potential direct and indirect effects of chemical pesticides on human health and beneficial insects, including natural enemies of pests (NE) that are biological control agents of insect pests via predation and parasitism [12,13,14]. The adoption of more sustainable farming practices will benefit both the environment and human health. One alternative is conservation biological control which can regulate arthropod NE populations through multitrophic interactions and a balance between pests and their NEs [15]. Conserving locally adapted NEs is cost-effective and relatively simple and is an important consideration in pest management decisions [16] and strategies such as engineering agroecosystems to provide extra resources that would be limited in field crops [17]. A key component of landscapes that support NE populations are non-crop habitats rich in plant biodiversity, such as field margins that offer nectar, pollen, shelter and alternative hosts to NE communities, and thus provide support to enhance their populations and enhance sustainable agricultural benefits [18,19,20,21,22,23,24]. The presence of non-crop habitats surrounding or within arable land has been associated with increasing arthropod NEs of pests by providing floral resources, thus sustaining their populations [25,26]. For instance, important NEs such as parasitoids use nectar to fulfil their nutrition requirements at some stages of their development while spiders, lady beetles, rove beetles, syrphid flies, true bugs and lacewings use non-crop habitats to provide them with refuge, alternative hosts, pupation and overwintering sites [25,26,27,28,29,30,31,32,33,34,35,36,37]. It has been found that populations of NEs in field crops decline as the distance from the field margin increases and this demonstrates the essential function of field margins in maintaining NEs [38]. Non-crop habitats, such as field margins are important during crop senescence as NEs move from field crops to other resources [39]. However, floral resources provide different benefits to specific taxa of NEs [40], and thus NEs would respond differently to the proportions of non-crop habitats. For instance, the abundance of carabids was observed to decrease with the increase of the proportion or the presence of non-crop habitats; however, populations of spiders were not affected by the proportion or presence of non-crop habitat [41,42]. Thus, semi-natural habitats at a landscape scale offer benefits to NEs [43,44], while establishing non-crop habitats such as field margins will benefit NEs at a local scale [45], to enhance the ecosystem service of biological control of pests in agricultural fields. Existing knowledge about how non-crop host plants support NE communities is insufficient in many cropping systems including beans but it is essential when planning conservation biological control interventions [15,46]. The provision of alternative habitat and plant resources to support increased NE populations is an approach to pest management that will likely be economically and environmentally sustainable for smallholder farmers in SSA because of the availability of plants at local scales [22,47]. The importance of agroecosystem diversity and abundance, particularly in the field margins, for arthropod NE communities in smallholder bean farming systems of SSA is poorly understood. In addition, some studies have shown that intercropping enhances NEs of pests more than monocropping systems in legumes [48,49] although this is also understudied in smallholder common bean growing systems of SSA. In this study we took the framework of the following assumptions around how field margins are expected to benefit the crop and tested how they affect NE populations:

1. Plant-rich field margins influence the number of NEs in bean fields
2. An increase in NEs assemblage in field margins influence their numbers within the crop.
3. Intercropping in bean fields is associated with high populations of NEs compared to monocropping.

## 2. Materials and Methods

### 2.1. Sampling Natural Enemies from the Fields

This field trial was carried out in Kwa Sadala Village in the Hai District, Kilimanjaro Region, Tanzania (3°10′0″ S, 37°10′0″ E). Thirty-two sites ≥0.20 ha with either high (n = 16) or low (n = 16) plant diversity were selected based on the observed number of plant species in non-crop vegetation around each farm [50]. To quantify the diversity of the plant species in our field sites, the Shannon index (H′) [51] was used according to the formula below to calculate low diversity fields (H′ = 1.2) and high diversity fields (H′ = 2.3).

Shannon Index (H′) = H = −∑(pi(ln pi)).

pi—Proportion (n/N) of individuals of particular species in a whole community,
n—individuals of a particular species,
N—total number of individuals found,
∑—Sum symbol,
ln = natural logarithm to base e.

The field margins were at least ≥2.5 m wide. The surrounding composition was similar in all fields (the arable fields, dominated by several flowering weed species) with similar management practices without chemical spray. Fields were located at least 50 m apart.

A further parameter was the inclusion of cropping practice where half of the farmer fields at each level (low and high plant diversity fields) practiced monocropping of common beans (*Phaseolus vulgaris*), whereas the other half of farmer fields intercropped beans with maize (*Zea mays* L.). Sweep netting was carried out, one replicate per site per visit, using a standard canvas hand sweep net to sample insects. Each sweep replicate consisted of three parallel transects in which the net was swept back and forth ten times: transect 1 along the margin, at least 0.5 m from the crop; transect 2 in the crop edge, 5 m from the margin, and transect 3 in the centre of the crop, >15 m away from the margin. The insects collected by sweep netting were transferred to 95 to 99% ethanol for preservation. This was repeated six times over the growing season, one time at seedling, two times at vegetative and flowering/pod formation, and one time at physiological maturity before pod drying. Yellow sticky traps that had glue on both sides measured 25 × 10 cm (Real IPM, Nairobi, Kenya) were placed in the field margins monthly from May to August, corresponding to the growth stages of the crop. Every two sticky cards in each of the field margins for thirty-two sites were attached at the height of approximately 1 m from the ground to a wooden cane with a string wire. The sticky cards were collected after 48 h [52]. Cards were brought to the laboratory, for isolation of NEs captured. The cards were examined under a dissecting microscope to record NEs [53] and then the insects were removed from the traps using soft and thin forceps [52]. The insects were preserved in 95 to 99% ethanol.

Insects collected were categorized into taxonomic groups: parasitic wasps (Hymenoptera: Ichneumonidae and Braconidae) including *Aphidius* spp.; predatory bugs (Hemiptera: Reduviidae and known predatory Pentatomidae); lady beetles (Coleoptera: Coccinellidae) including *Cheilomenes lunata*; lacewings (Neuroptera: Chrysopidae) including *Chrysoperla congrua*; parasitic flies (specifically Diptera: Tachinidae); hoverflies (Diptera: known aphidophagous (Syrphinae) Syrphidae only, and excluding *Eristalini* species with aquatic larvae); predatory flies (specifically Diptera: Dolichopodidae and Asilidae with predatory adults); and all other predatory beetles (Coleoptera: known predatory Carabidae, Lycidae and Staphylinidae). Specimens were identified to the highest level of resolution possible but focused on characterising them by life history and functional groups. We categorised the Pentatomidae, Carabidae and Syrphidae into “known predators” and analysed only these data.

### 2.2. Estimation of Aphid Severity

The severity of *A. fabae* infestation was estimated using a visual rating of 1–6, where: 1 = no aphids; 2 = 1–100 aphids; 3 = 101–300 aphids; 4 = 301–600; 5 = 601–1000 and 6>1000 aphids as used previously [54] from ten randomly selected bean plants in each field weekly throughout the crop development stages.

### 2.3. Statistical Analysis

We used the generalized linear models (GLM) procedure assuming Poisson distribution with log link function to compare the number of NEs (dependent variable) among high and low-diversity fields, location in the fields, months and cropping systems (explanatory variables) [55]. The model with the best fit was selected using the Akaike Information Criterion (AIC) and Bayesian Information Criteria (BIC) tests [56], whereas the model with the lowest AIC and BIC values was selected. The Shapiro-Wilk test was used to check for normality (SPSS Version 22.0). We estimated the overdispersion parameter by Pearson chi-square divided by degrees of freedom and estimated by maximum likelihood [44]. Pairwise comparisons were done with the Holm multiple comparisons test in the ‘emmeans’ package in (RStudio Version 1.2.1335) [57].

## 3. Results

### Spatial and Temporal Distribution of Natural Enemies in Bean Fields

**Figure 1:**
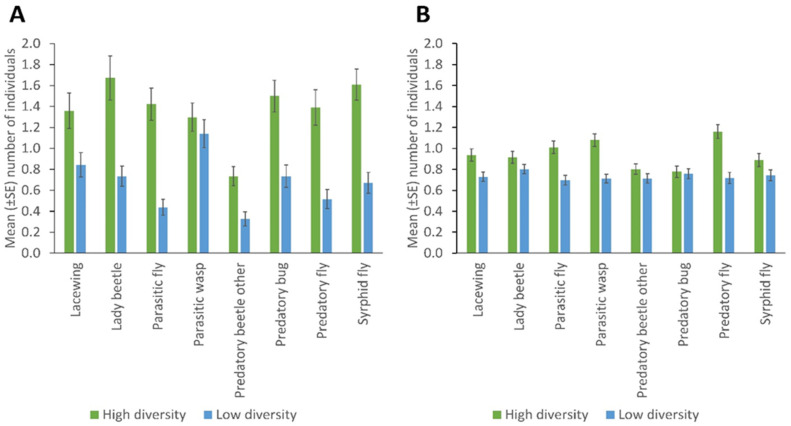
Natural enemies’ abundance in fields with high and low plant diversity margins, collected by (**A**) sticky trapping and (**B**) sweep netting (Error bars = s.e.m.)

With the sticky trapping, the most abundant taxa were parasitic wasps (Ichneumonidae and Braconidae), with the Braconidae (particularly *Aphidius colemani*) being the dominant family) while with the sweep netting the most abundant taxa were the predatory flies (Dolichopodidae and Asilidae) with the Dolichopodidae being the dominant family in the study. The hymenopteran taxonomic data were obtained from a parallel study using mitochondrial cytochrome oxidase I barcoding of insects collected from sentinel plants, showing the common hymenopteran groups present in the study area [58]. The high-diversity fields had a significantly higher number of lady beetles, predatory flies, hoverflies, predatory bugs, parasitic flies, other predatory beetles (*p* = 0.001), and lacewings (*p* = 0.005), caught through sticky trapping used to monitor the field margins for NEs, than the fields with low diversity. No significant differences were observed in the number of parasitic wasps between high and low-diversity fields (Figure 1A; Supplementary Table S1).

**Figure 2:**
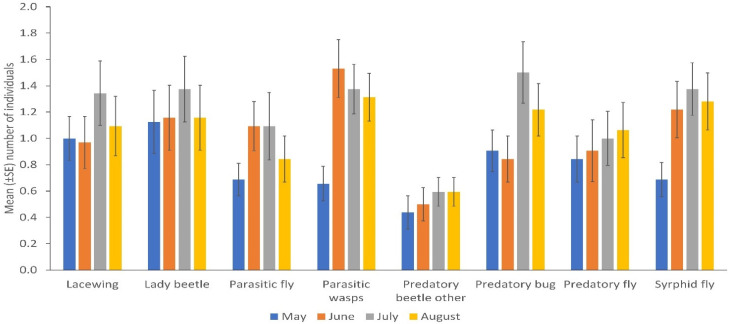
Natural enemies’ distribution in different months collected by sticky trapping. May corresponds to the late seedling and vegetative stage; June corresponds to the flowering stage; July corresponds to the fruiting stage and early maturity stages and August corresponds to the late maturity stage of the crop near to harvest (Error bars = s.e.m.).

**Figure 3:**
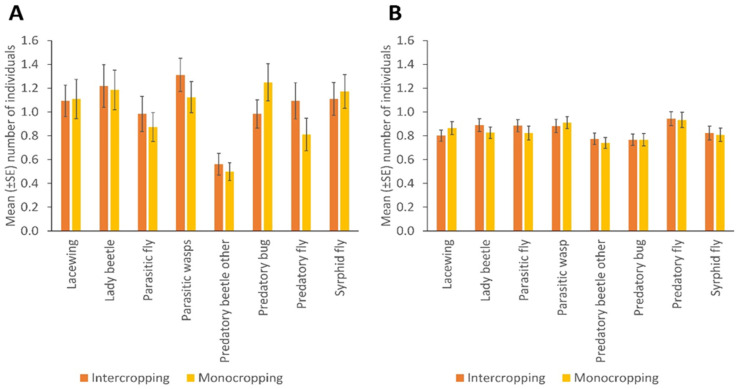
The number of natural enemies in bean monocropped and intercropped fields, collected by (**A**) sticky trapping and (**B**) sweep netting (Error bars = s.e.m.).

NE populations differed over the duration of the experiment in terms of catches of parasitic wasps (*p* = 0.004) and syrphid flies (*p* = 0.031) by the sticky trapping. There were no differences in the number of lady beetles, predatory flies, parasitic flies and other predatory beetles in different months. Parasitic wasps and syrphid flies were significantly more numerous in the flowering stage of the crop (*p* = 0.001; *p* = 0.032); fruiting stage and early maturity stages of the crop (*p* = 0.005; 0.008); and late maturity stage of the crop (*p* = 0.009; 0.018) than in the late seedling and vegetative stages of the crop, respectively. Predatory bugs were significantly more frequent in the fruiting stage and early maturity stages of the crop (*p* = 0.032) than in the flowering stage of the crop (Figure 2; Supplementary Table S3). More parasitic wasps (*p* = 0.001), lacewings (*p* = 0.006), syrphid flies (*p* = 0.009), parasitic flies (*p* = 0.001) and predatory flies (*p* = 0.001) were caught via sweep netting from high plant diversity fields compared to fields with low plant diversity in margins, but other insect taxa did not differ in abundance according to margin type (Figure 1B; Supplementary Table S2). No significant differences were observed between cropping systems (mono-cropping versus intercropping) for both sticky trap and sweep netting collections (Figure 3A,B).

**Figure 4:**
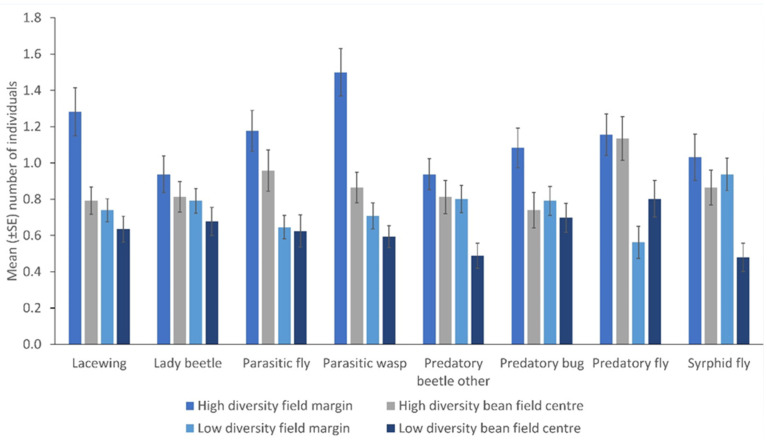
The number of natural enemies in the field margin and the centre of the fields from low and high plant diversity fields as sampled by sweep nets (Error bars = s.e.m.).

There were more NEs in the margin relative to the crop edge (lacewings, *p* = 0.046; parasitic wasps, *p* = 0.041; predatory bugs, *p* = 0.004), except for syrphid flies, other predatory beetles, parasitic flies and predatory flies. Moreover, there were more insects in the margin relative to the centre of the field (parasitic wasps, *p* = 0.001; syrphid flies, *p* = 0.002; lacewings, *p* = 0.005; other predatory beetles, *p* = 0.043) except for parasitic flies, predatory bugs, and predatory flies (Table 1). There were few consistent differences in the number of NEs within fields but consistently higher counts from high plant diversity fields were observed (other predatory beetles, *p* = 0.008; parasitic wasps, *p* = 0.001; predatory fly, *p* = 0.046; syrphid fly, *p* = 0.001) (Figure 4).

Plant diversity significantly influenced the abundance of all NE groups caught by sticky trapping (lady beetles, predatory flies, hoverflies, predatory bugs, parasitic flies, other predatory beetles, *p* = 0.001; lacewings, *p* = 0.005), except for parasitic wasps; while, with sweep netting, the plant diversity had significant effects on the abundance of parasitic wasps, parasitic flies, predatory flies (*p* = 0.001), lacewings (*p* = 0.006), and syrphid flies (*p* = 0.009), except lady beetles, predatory bugs, other predatory beetles. Cropping systems had no significant influence on any of the NE groups. Parasitic wasps’ and syrphids’ abundance were influenced significantly by the time of sampling (*p* = 0.004; *p* = 0.031 respectively), while lady beetles, predatory flies, predatory bugs, parasitic flies and other predatory beetles’ abundances were not affected. Syrphids, lacewings and predatory bugs were influenced significantly by field location (*p* = 0.001; *p* = 0.010; *p* = 0.027 respectively) except for other predatory beetles, lady beetles, predatory flies, parasitic flies and parasitic wasps.

**Figure 5:**
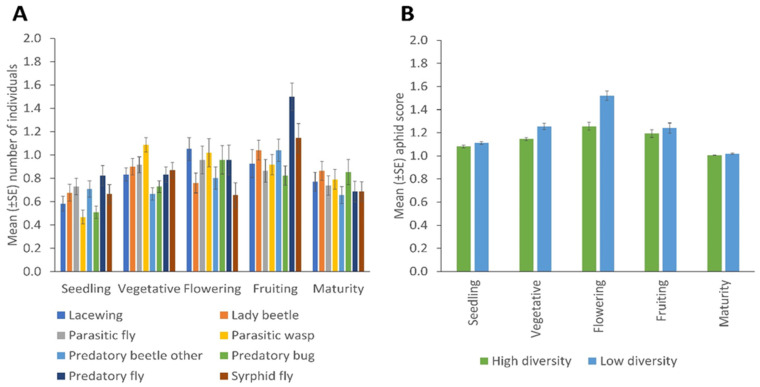
Changes across the crop’s development stages in (**A**) the number of natural enemies (collected by sweep netting) of the various taxa, and (**B**) aphid infestation score, according to margin plant diversity (Error bars = s.e.m.).

Significant effects of the crop development stage on the distribution of NEs for sweep netting collections were observed for lady beetles (*p* = 0.039); other predatory beetles (*p* = 0.020); syrphid flies (*p* = 0.001) and predatory flies (*p* = 0.001) (Figure 5A). There were no significant differences in the mean number of aphids (*A. fabae*) observed among different crop development stages and between high and low plant diversity fields. The *A. fabae* population was high in the flowering stage compared to other crop developmental stages and in low fields compared to the high plant diversity fields (Figure 5B).

## 4. Discussion

The classic assumptions of conservation biological control are that flowering plants and an abundance of non-crop habitat near a crop will enhance populations of NEs, that the NEs will move into the crop from this habitat, and that those NEs will eat or parasitise pests of the crop, resulting in better pest management and ultimately reduced yield losses. We set out to test these assumptions by comparing smallholder farms with different levels of plant diversity in field margins in terms of the populations of NEs they support and then evaluating whether those predatory NEs controlled key pests within the cropping area.

NEs that were collected in bean fields included predatory bugs, lacewings, predatory flies, parasitic flies, parasitic wasps, lady beetles and diverse other predatory beetles. In accordance with the expectations of conservation biocontrol theories, plant diversity in field margins had a positive impact on the number of NEs but notably, this showed up in the sticky trap data more than in the sweep netting data. This suggests that either method alone may not give an accurate indication of NEs; sticky traps may oversample volant insects relative to non-volant insects (including larvae), indicating that whether or not populations were higher in the rich margins, flight activity (implying perhaps movement within the crop) may have benefitted from richer margins. In no cases did the rich margins reduce populations of any NE taxa. Sweep netting and sticky traps have been used and are common for the collection of NEs. Sticky traps and sweep nets, for instance, have been used to collect different taxa of NEs (parasitoids, lady beetles, hoverflies, true bugs and lacewings) [35,52,53,59,60,61].

Our findings that field margins promote NE activity and/or populations concur with other studies on smallholder farms and studies such as Arnold et al. [19] and Mkenda et al. [22], which found a strong association between flower strips and plant-rich patches with NE communities. As reported by Rebek et al. [52], we found that parasitic wasps were the most abundant of the groups studied and that highly mobile individuals, such as parasitic flies, syrphid flies, and lady beetles, were caught in large numbers by sticky cards [52]. (Figure 1A; Supplementary Table S1). With the yellow sticky traps, different insect behaviours might have affected the number of NEs caught [53]. For example, yellow traps were more likely to trap Hymenoptera and Diptera, whereas blue is favoured by Thysanoptera [62,63]. There was no significant difference in the number of parasitic wasps collected through sticky trapping, and no significant differences observed in the number of lady beetles, predatory bugs, and other predatory beetles collected through sweep netting, between high and low plant diversity fields. These NEs might have been influenced by other factors like the presence of host (aphids) in the field crop [64]. Other between-site differences in NE populations, even where the plant abundance was similar, may be explained by wider differences in field management [65]. Disturbances, such as pesticide applications and cutting, have impacts on the activities of NEs and could affect populations of prey for NEs [66].

Higher numbers of parasitic wasps (from both sweep netting and sticky trapping), syrphid flies (sweep netting), syrphid flies (sticky trapping), and predatory bugs in July mostly corresponded to the bean flowering stage and changes in the frequency of catching of lacewings (sweep netting) might be due to the biotic and abiotic factors contributing to seasonal dynamics in arthropod abundance [67,68]. NE communities respond to environmental factors differently [69]. The variations may also be explained by changing prevailing environmental conditions, for example, an increase in floral resources towards the flowering stage of the bean crop. Our work adds to existing research findings showing a high abundance of NEs is associated with the provision of floral resources from plants [52,67,70,71,72,73,74]. Including a mixture of plants in agricultural systems can provide varied and complementary resources that play specific roles to NEs [75]. NEs depend on other local and landscape characteristics such as fertilizer and pesticide application, crop rotation, tillage practices, and the composition of the field surroundings [38,60,76,77]. Some studies have shown the negative effects of chemical pesticide application on the NEs of pests. Thus field margins can be used to mitigate the negative effects of insecticides on populations of NEs [78,79,80,81,82,83,84,85,86,87]. Lethal and non-lethal effects such as mortality and feeding deterrents on NEs have been associated with the application of chemical pesticides [81,82,83,84,85,86]. Generally, there was an increase in mean aphid populations around the flowering stage of the bean crop and this might have corresponded to the availability of quality host plants, although no significant differences were observed among different crop stages. High populations of aphids have been observed in the flowering stage by Azimi and Amini [88]. The study by Birch [89] found the lowest *A. fabae* populations in the crop maturity stage, probably due to older plants that are lower quality hosts and also due to increased predation/parasitism by NEs. The survival and reproduction of aphids depend on high-quality hosts for food sources [90].

For most insect taxa, we found consistently higher numbers in the margin relative to the crop. This agreed with most other studies, showing limited movement into the crop of insects with margin-based communities [22,91,92,93,94,95]. However, a few taxa also occurred in high numbers in the centre of the field including other predatory beetles, parasitic wasps, predatory flies and syrphid flies; as these readily enter the crop, they could be an ideal focus for future biocontrol research.

We saw a subtle effect of intercropping versus monocropping on the natural enemy numbers: while overall populations were higher in intercropped systems, no individual taxon was more abundant in intercropped fields. Parasitic wasps, for instance, come out higher in intercrops on sticky traps but their numbers are high in monocrops with sweep nets. Thus, with no consistent patterns, the effects of mono v intercropping were not significant. A few studies have found populations of NEs enhanced through intercropping [88,96]. However, based on our evidence intercropping alone as a method to support NE populations may not yield improved pest management benefits and needs to be combined with other agroecological interventions. NEs have been associated with the field margins and non-crop habitats for resources such as pollen and nectar. In addition, these habitats may offer alternative prey, corridors for their dispersal and places for overwintering and reproduction [19,22,52,92,97,98,99,100,101]. Thus, with habitat disturbances and loss due to agricultural intensification, field margins could play a key role in conserving NE communities and consequently, enhancing biological control of pests in bean fields, especially for resource-constrained smallholder farmers [18].

## 5. Conclusions

Field margins are valuable in minimizing the negative impacts of agricultural intensification on NE populations; therefore, bringing resilience at local and landscape scales. The abundance of plants within field margins can provide a wide range of seasonal resources to NEs. These resources may enhance NEs’ survival, longevity, and fecundity, and in turn, facilitate them in providing pest suppression. We found evidence that lacewing and lady beetle larvae and adult assassin bugs were consuming the major crop pest in this case; these should be a primary focus of future biological conservation efforts in these agricultural systems.

## Figures and Tables

**Table 1:** Mean ± (SEM) numbers of natural enemies in different field locations collected by sweep netting.

Field Location	Mean Number of Natural Enemies (±SEM)							
	Lady Beetle	Syrphid Fly	Lacewing	Parasitic Wasp	Predatory Fly	Parasitic Fly	Other Predatory Beetles	Predatory Bug
Field margin	0.86 ± 0.06 a	0.99 ± 0.08 a	1.01 ±0.08 a	1.10 ± 0.08 a	0.86 ± 0.08 a	0.91± 0.07 a	0.87 ± 0.06 a	0.94 ± 0.07 a
Crop edge	0.96 ± 0.07 a	0.79 ± 0.07 b	0.78 ±0.06 b	0.85 ± 0.06 b	0.99 ± 0.07 a	0.86± 0.06 a	0.75 ± 0.06 a	0.65 ± 0.05 b
Field centre	0.74 ± 0.06 a	0.67 ± 0.06 b	1.71 ±0.05 b	0.73 ± 0.05 b	0.97 ± 0.08 a	0.79± 0.07 a	0.65 ± 0.06 b	0.72 ± 0.06 ab

Values followed by the same letters (a and b) within the column are not significantly different (*p* < 0.05).

## Data Availability

Data is contained in supplementary material.

## Supplementary Materials

The following supporting information can be downloaded at: https://www.mdpi.com/article/10.3390/insects13070569/s1, Table S1. Mean ± (SEM) numbers of natural enemies in fields collected by sticky traps. Table S2. Mean ± (SEM) numbers of natural enemies in fields collected by sweep nets. Table S3. Mean ± (SEM) numbers of natural enemies in fields collected by sticky traps.
