# Supplementary material for: Plant-Rich Field Margins Influence Natural Predators of Aphids More Than Intercropping in Common Bean
Source: Insects. 2022 Jun 23;13(7):569. doi: 10.3390/insects13070569 (PMC9322975; doi:10.3390/insects13070569)
Supplement: Supplementary file 1 [file insects-13-00569-s001.zip › insects-1704218-supplementary.pdf]

Supplementary Tables

Table S1. Mean ± (SEM) numbers of natural enemies in fields collected by sticky traps.

Diversity	Mean Number of Natural Enemies (±SEM)							
	**Lady Beetle**	**Syrphid Fly**	**Lacewing**	**Parasitic Wasp**	**Predatory Fly**	**Parasitic Fly**	**Other Predatory Beetles**	**Predatory bug**
High	1.67 ± 0.21a	1.61 ± 0.17a	1.36 ± 0.17a	1.30 ± 0.14a	1.39 ± 0.17a	1.42± 0.15a	0.73± 0.09a	1.50± 0.15a
Low	0.73 ± 0.96b	0.67 ± 0.09b	0.84 ± 0.12b	1.14 ± 0.14a	0.51 ± 0.09b	0.44± 0.08b	0.33± 0.07b	0.73± 0.12b

Values followed by the same letters (a and b) within the column are not significantly different ($p < 0.05$).

Table S2. Mean ± (SEM) numbers of natural enemies in fields collected by sweep nets.

Diversity	Mean Number of Natural Enemies (±SEM)							
	**Lady Beetle**	**Syrphid Fly**	**Lacewing**	**Parasitic Wasp**	**Predatory Fly**	**Parasitic Fly**	**Other Predatory Beetles**	**Predatory bug**
High	0.90 ± 0.06a	0.89 ± 0.06a	0.90 ± 0.06a	1.08 ± 0.06a	1.16 ± 0.17a	1.01± 0.06a	0.80 ± 0.05a	0.78 ± 0.05a
Low	0.80 ± 0.04a	0.74 ± 0.05b	0.73 ± 0.04b	0.71 ± 0.04b	0.72 ± 0.05b	0.70± 0.05b	0.71± 0.05a	0.76 ± 0.05a

Values followed by the same letters (a and b) within the column are not significantly different ($p < 0.05$).

Table S3. Mean ± (SEM) numbers of natural enemies in fields collected by sticky traps.

Month	Mean Number of Natural Enemies (±SEM)							
	**Lady Beetle**	**Syrphid Fly**	**Lacewing**	**Parasitic Wasp**	**Predatory Fly**	**Parasitic Fly**	**Other Predatory Beetles**	**Predatory bug**
May	1.22 ± 0.24a	0.69 ± 0.13a	1.00 ±0.17a	0.66 ± 0.12a	0.84 ± 0.18a	0.69± 0.12a	0.44 ± 0.13a	0.91 ± 0.16ab
June	1.16 ± 0.25a	1.22 ± 0.21b	0.97 ±0.20a	1.53 ± 0.22b	0.91 ± 0.24a	1.09± 0.19a	0.50 ± 0.13a	0.84 ± 0.18a
July	1.38 ± 0.25a	1.38 ± 0.20b	1.34 ±0.24a	1.38 ± 0.19b	1.00 ± 0.21a	1.09± 0.26a	0.59 ± 0.11a	1.50 ± 0.23b
August	1.16 ± 0.25a	1.28 ± 0.22b	1.09 ± 0.23a	1.31 ± 0.19b	1.06 ± 0.21a	0.84 ± 0.18a	0.59 ± 0.11a	1.22 ± 0.20ab

Values followed by the same letters (a and b) within the column are not significantly different ($p < 0.05$)

May corresponds to the late seedling and vegetative stage; June corresponds to the flowering stage; July corresponds to the fruiting stage and early maturity stages and August corresponds to the late maturity stage of the crop near harvest
